# Diffuse Large B-Cell Lymphoma Revealed by Splenic Abscess: A Case Report

**DOI:** 10.7759/cureus.18771

**Published:** 2021-10-14

**Authors:** Soufiane Taibi, Rachid Jabi, Yassin Kradi, Nadir Miry, Mohammed Bouziane

**Affiliations:** 1 Department of General Surgery, Mohamed VI University Hospital, Faculty of Medicine and Pharmacy, Labarotory of Anatomy, Microsurgery and Surgery Experimental and Medical Simulation, Mohammed First University, Oujda, MAR; 2 Department of Pathology, Mohammed VI University Hospital, Faculty of Medicine and Pharmacy, Mohammed First University, Oujda, MAR

**Keywords:** ps-dlbcl, splenectomy, abscess, spleen, dlbcl

## Abstract

Diffuse large B-cell lymphoma (DLBCL) is one of the most common non-Hodgkin lymphomas. It has no typical or specific clinical features. DLBCL revealed by an abscess is a rare entity. CT is sensitive in detecting splenic abscesses, and it can define the exact location and extent of the abscess as well. The splenic abscess is associated with typhoid fever, AIDS, abdominal infections, pneumonia, bacterial endocarditis, and urogenital infections, parasitic abscesses, organ transplantation, or neoplastic diseases. DLBCL is not usually related to its etiology. Elective open splenectomy, both diagnostic and therapeutic, is the gold standard method of management today and has low morbidity and mortality rates, with even lower rates for laparoscopic splenectomy. The diagnosis of DLBCL is based on the anatomopathological and immunohistological examination. We report a case of a man with a splenic abscess initially treated as an abscess of bacterial origin; however, the lack of improvement in his condition led us to perform a splenectomy, and the anatomopathological study revealed a DLBCL.

## Introduction

Diffuse large B-cell lymphoma (DLBCL) is the most common non-Hodgkin's lymphoma and comprises a large number of different entities with different clinicopathological features. It accounts for approximately 30% of all adult lymphomas [1]. Due to the functional heterogeneity of lymphoid cells and their ubiquitous anatomical distribution, these diseases can develop in any organ or tissue of the body. There are no typical or specific clinical features associated with the condition; however, DLBCL revealed by an abscess is a rare occurrence [2].

DLBCL is one of the so-called "aggressive" lymphomas, which progresses spontaneously, but is characterized by high chemosensitivity [3]. Spleen abscesses are usually related to infectious diseases, but a lymphomatous origin must also be considered. We present an unusual case of DLBCL in a 43-year-old man revealed by a splenic abscess and discuss the diagnostic and therapeutic modalities.

## Case presentation

The patient was a 43-year-old male with a surgical history of the resection of a prominent dome of a hepatic hydatid cyst one year prior to the admission. He had been referred to the emergency department for the management of acute abdominal pain located in the left hypochondrium that had been evolving for two weeks and associated with multiple episodes of vomiting without any other signs, as well as with fever and altered general condition (loss of 10 kg of body weight in one month). On physical examination, the patient was conscious, hemodynamically and respiratory-wise stable, and anicteric. His abdomen was not distended, and the presence of a median laparotomy scar was observed, along with left hypochondrial tenderness and painful splenomegaly measuring 13 cm on palpation; no adenopathy was noted. Laboratory studies indicated hyperleukocytosis (26,570/mm^3^), elevated inflammatory markers, C-reactive protein (CRP) of 202 mg/dl, hypochromic anemia [hemoglobin (Hb) of 8.6 g/dl], and elevated lactate dehydrogenase LDH level. The rest of the laboratory tests were normal, especially HIV serology, hydatid serology, and tumor markers (carcinoembryonic antigen and cancer antigen 19-9).

**Figure 1:**
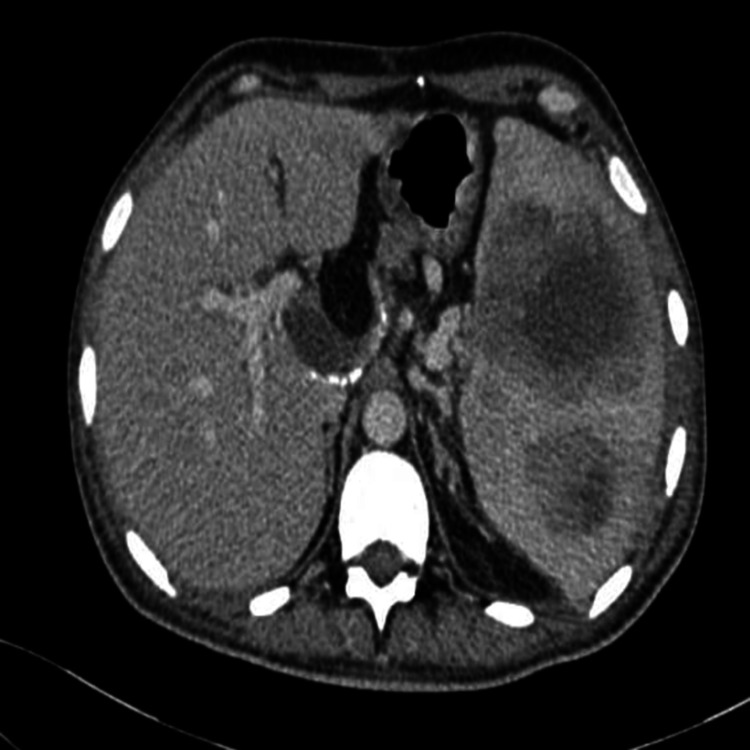
CT axial section showing a splenic abscess CT: computed tomography

An abdominal CT scan showed an enlarged spleen measuring 168 mm, heterogeneous, with multiple hypodense areas, some of which contained a fluid component, the largest one measuring 65 x 57 mm at the medio-splenic level, related to abscesses associated with peri-splenic fat infiltration (Figure 1).

**Figure 2:**
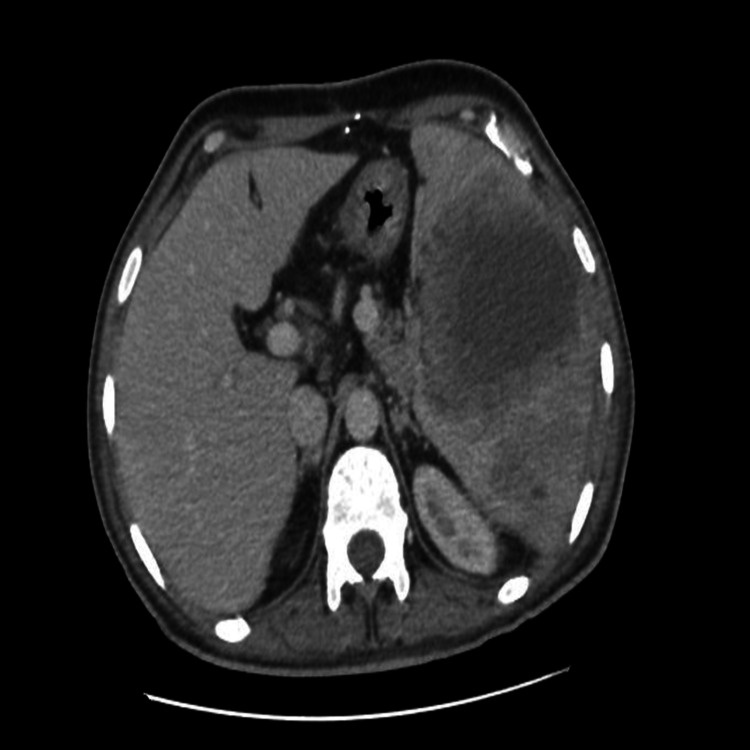
Control CT axial section after antibiotic therapy showing a stable aspect of the splenic abscess CT: computed tomography

The patient received third-generation cephalosporin and metronidazole antibiotics for seven days. However, his symptoms persisted and all biological studies were unchanged; a CT scan was performed, which showed an increase in the number and size of multiple abscesses with thickened and enhanced walls after contrast injection, the largest one measuring, at the medio-splenic level, 111 x 94 x 84 mm vs. 65 x 57 mm on the first imaging (Figure 2).

**Figure 3:**
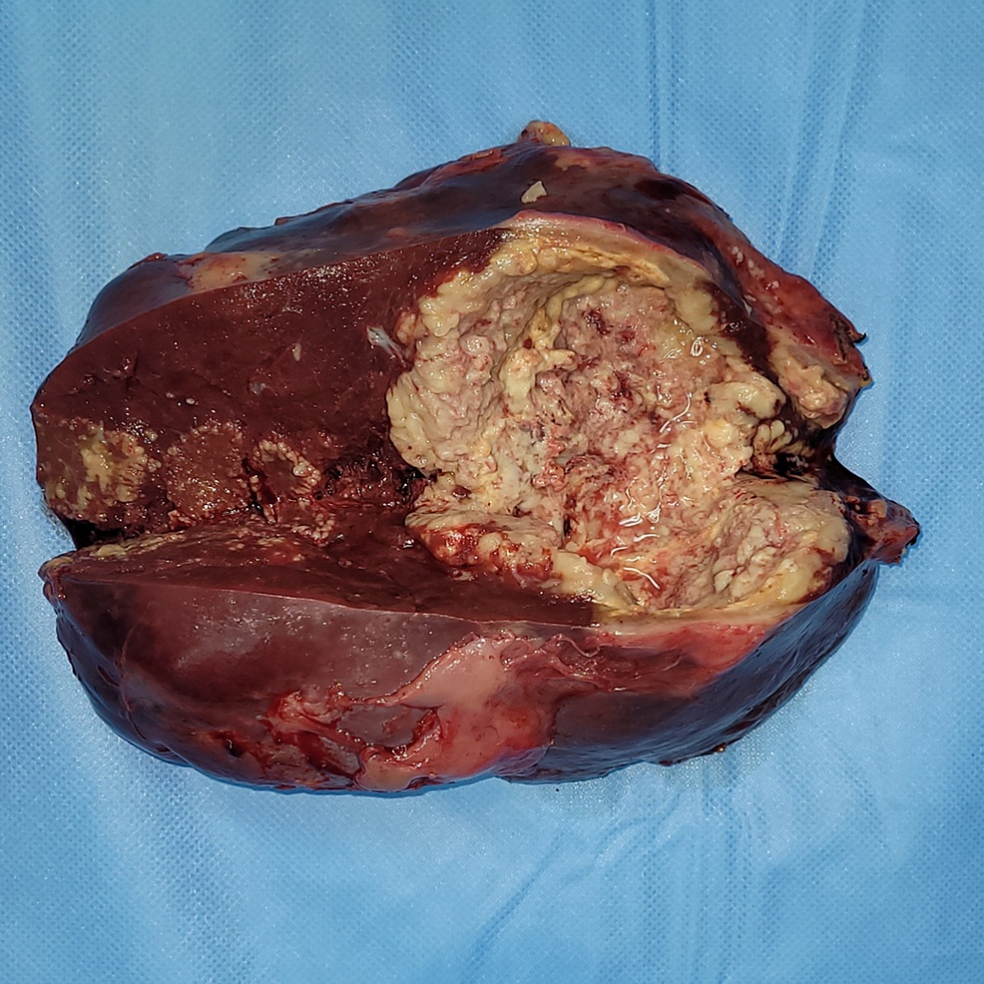
Total splenectomy showing an abscess at section

He underwent an exploratory midline laparotomy; on surgical exploration, splenomegaly containing an intraparenchymal abscess plugged by the omentum and transverse colon was found. A total splenectomy with abdominal drainage was performed (Figure 3).

Postoperatively, the patient received analgesia, prophylaxis for thromboembolism, and ceftriaxone (2g IV daily) with metronidazole 500 mg IV (every eight hours); oral diet was started on postoperative day one. The postoperative recovery was uneventful. The drain was removed and the patient was discharged on day four.

Macroscopic examination of the resected specimen revealed a spleen measuring 20 x 16 x 4.5 cm. On section, multiple well-limited abscessed nodules with pus discharge measuring between 8 x 4.5 cm and 1.3 x 1.2 cm were found.

Histologically, systematic sampling of the abscessed areas showed ulcerated splenic parenchyma widely infiltrated by a malignant tumor proliferation arranged in diffuse layers. It consisted of large, atypical cells with hyperchromatic nucleoli surrounded by clear cytoplasm. Several patterns of mitosis were noted with a fibroinflammatory stroma.

**Figure 4:**
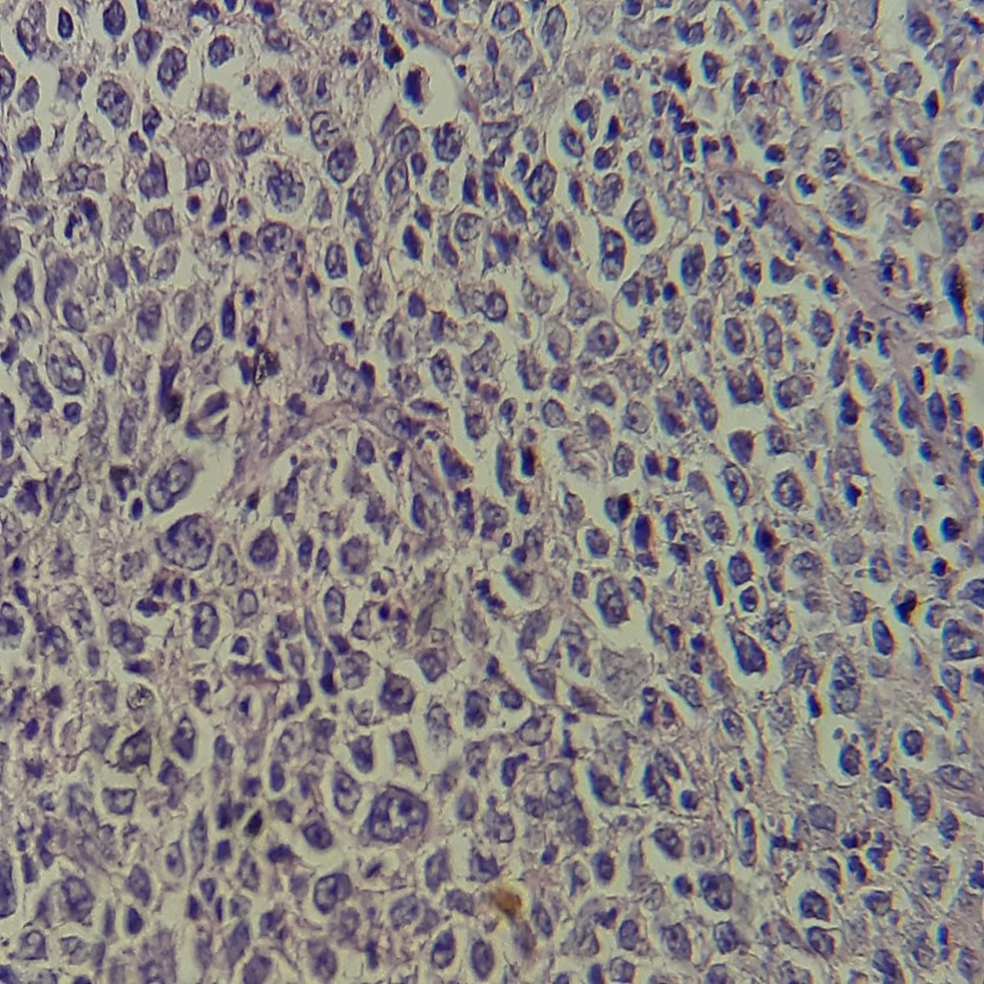
Low magnification view showing a diffuse proliferation of large medium-sized lymphoid cells with large, rounded nuclei containing one or more nucleoli and a scant lymphophilic cytoplasm; numerous mitoses can be seen (H&E stain)

**Figure 5:**
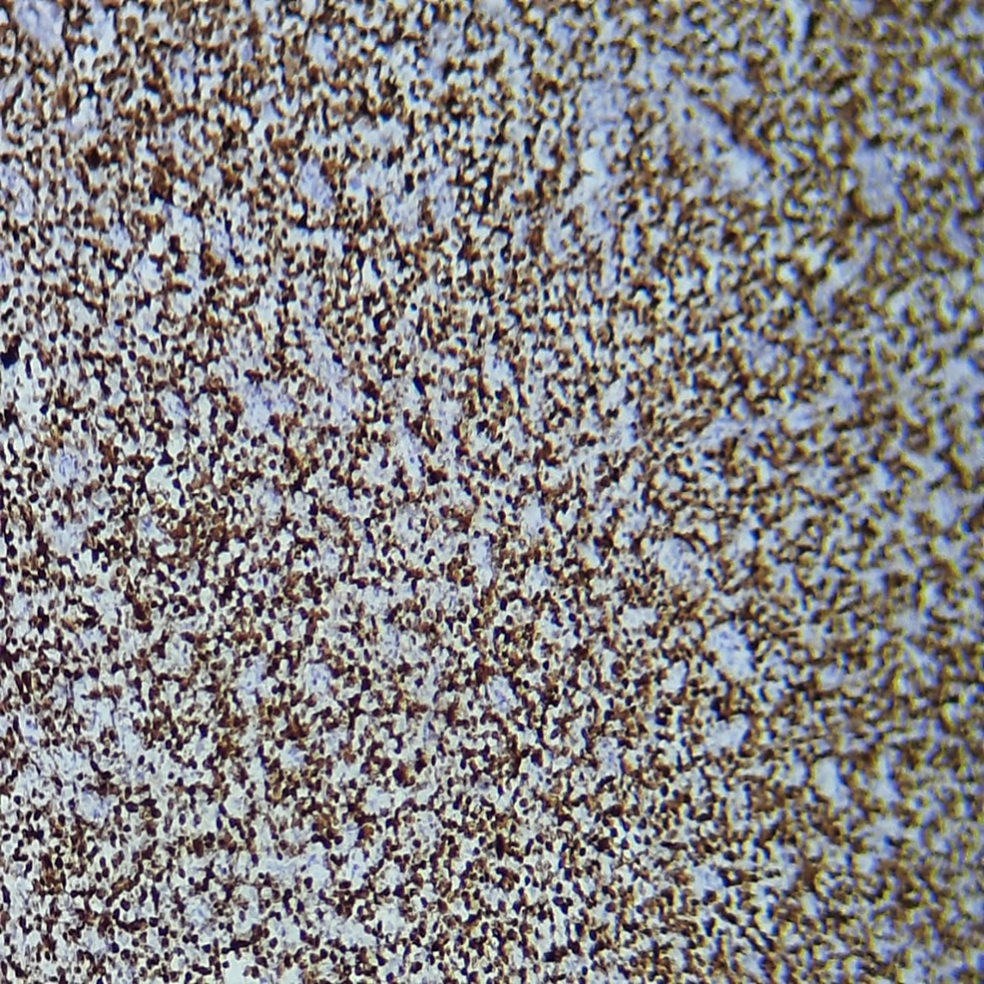
The proliferation shows strong and diffuse positive cytoplasmic staining with CD20 and high positive nuclear staining with Ki-67 (90%) - image 1

**Figure 6:**
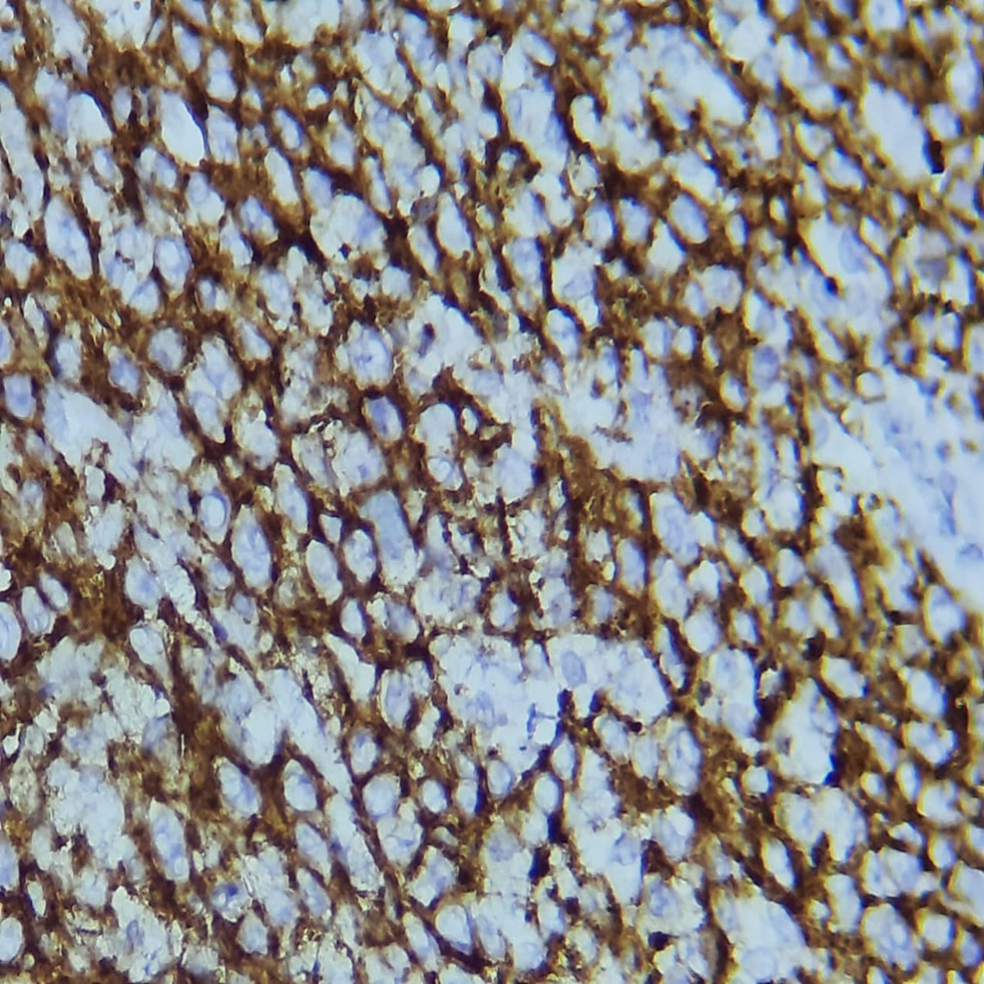
The proliferation shows strong and diffuse positive cytoplasmic staining with CD20 and high positive nuclear staining with Ki-67 (90%) - image 2

The immunohistochemical study showed diffuse positive labeling of tumor cells by CD20, PAX5, and CD30, reactive T cells labeled by CD3, nuclear labeling by BCL6, focal labeling by BCL2, and absence of labeling of tumor cells by CD15, CD10, EMA, and ALK. The Ki67 proliferation index was estimated at 90%. On the basis of these histological and immunohistochemical aspects, the diagnosis of DLBCL was established (Figures 4, 5, 6).

The patient was referred to the internal medicine department for further management, including additional immunohistochemistry by multiple myeloma oncogene 1 (MUM1) to clarify the germinal center or non-germinal center profile.

After the diagnosis of DLBCL was made, it was decided to transfer the patient to the internal medicine department, where he underwent a pre-chemotherapy workup and then began treatment as soon as possible.

## Discussion

Although DLBCL is the most frequent pathological variety among lymphoid hemopathies [1] and accounts for approximately 30% of all adult lymphomas, it remains the rarest entity among splenic non-Hodgkin lymphoma types [4]. Primary splenic diffuse large B-cell lymphoma (PS-DLBCL) is very rare and accounts for only 1% of malignant lymphomas [5], for which there is no precise definition or classification as hematolymphoid neoplasia [6]. The definition of PS-DLBCL is still very controversial. For example, when Dasgupta et al. [7] represented it as neoplasia of the spleen and its approximate nodes, Kehoe and Straus indicated that other lymph nodes could be involved [8], and other hypotheses suggest that the involvement of the spleen is a primary criterion for PS-DLBCL, considered as an advanced lymphoma [4,9-10]. Pathologically, PS-DLBCL is localized in the splenic white pulp, which usually forms a large nodule or mass [11]. PS-DLBCL may extend directly beyond the splenic capsule and invade adjacent organs, such as the pancreas, stomach, diaphragm, colon, and omentum.

DLBCL revealed by splenic abscess is rare. In a study conducted on the management of splenic abscess involving 16 cases, the results regarding the causes of splenic abscess were mainly related to typhoid fever; nowadays, the most common causes are AIDS, abdominal infections, pneumonia, bacterial endocarditis, urogenital infections [12], parasitic abscesses, organ transplantation, or neoplastic diseases; DLBCL is not related to the etiology [13].

The clinical presentations of PS-DLBCL are usually variable and nonspecific; the most common symptoms are abdominal pain, probably due to the enlarged spleen, weight loss, fever, night sweats, and the presence of one or more splenic masses. In our case, the most frequent symptoms were abdominal pain, fever, and a large splenic abscess. These findings are in agreement with the study by Bairey et al. [5]. CT is sensitive in detecting splenic abscesses, and it can help define the exact location and extent of the abscess better than ultrasound and the presence of subcapsular and per splenic pathology [14]. Diagnostic options include ultrasound-guided splenic biopsy and fine-needle aspiration, which have been shown to have good diagnostic accuracy and low morbidity [4].

The definitive diagnosis of DLBCL is based on pathological examination. Morphological examination shows diffuse proliferation of large B cells, with nucleoli at least twice the size of a small lymphocyte [15]. At the immunohistochemical level, the markers necessary for the diagnosis of DLBCL are CD20, which mark B lymphocyte differentiation, CD79a, and PAX5. Intracytoplasmic immunoglobulins are required in cases of plasma cell differentiation, as CD20 and CD79a are negative. Ki67 reflects cell proliferation. The markers CD10, BCL6, and MUM1 constitute the Hans algorithm [16]. MYC and BCL2 antibodies can also provide relevant information. CD5 is essential because it can provide a differential diagnosis of mantle cell lymphoma if cyclin D1 is also positive. Germinal center B-cell (GCB) lymphomas have the gene expression profile of germinal center B-cells and have a better prognosis than activated peripheral B-cell (ABC) lymphomas [15].

Splenectomy in PS-DLBCL has therapeutic and diagnostic purposes although they have not yet been fully elucidated. If performed at an early stage, it significantly increases overall survival (OS) and progression-free survival (PFS) [5] rates, but no proven recommendations for aggressive lymphomas have been published [5]. In our case, DLBCL presented clinically as a spleen abscess, and hence we used splenectomy for diagnostic and therapeutic purposes. However, splenectomy performed at an early stage of PS-DLBCL also has a therapeutic impact and significantly improves OS and PFS [5].

Adjuvant therapy for DLBCL has improved markedly, largely due to a better understanding of the biology and molecular pathways of the disease. The addition of rituximab to standard CHOP chemotherapy has dramatically improved response rates and survival in all categories of DLBCL patients [17].

An elevated LDH level is a poor prognostic factor even in the rituximab era [18]. Tumor staging plays a crucial role in the prognosis and management of splenic lymphomas. According to Ahmann et al., splenic lymphomas are classified into three stages: stage I, which corresponds to strict spleen involvement; stage II, which corresponds to hilar lymph node involvement; and stage III, which corresponds to extra-splenic involvement [19]. Accordingly, our case was classified as stage I.

## Conclusions

DLBCL is the most common lymphoma type in adults. These lymphomas are clinically, morphologically, phenotypically, and molecularly heterogeneous. The splenic DLBCL revealed by a spleen abscess remains a rare entity, and in such cases, the role of the biological markers and CT, diagnostic and therapeutic splenectomy, and finally the histological examination of the specimen is critical to confirm the diagnosis. This case report, which discussed an isolated DLBCL of the spleen revealed by an abscess, contributes to the understanding of this condition.
